# Predicting the Outcomes of Combination Therapy in Patients With Chronic Hepatitis C Using Artificial Neural Network

**DOI:** 10.5812/hepatmon.17028

**Published:** 2014-06-01

**Authors:** Forough Sargolzaee Aval, Nazanin Behnaz, Mohamad Reza Raoufy, Seyed Moayed Alavian

**Affiliations:** 1 Faculty of Medicine, Shahid Beheshti University of Medical Sciences, Tehran, IR Iran; 2 Department of Physiology, Faculty of Medical Sciences, Tarbiat Modares University, Tehran, IR Iran; 3 Middle East Liver Diseases Center (MELD), Tehran, IR Iran

**Keywords:** Cholesterol, Hepatitis C, Chronic, Hemoglobin, IL28B Protein, Human, Peginterferon Alfa-2b, Ribavirin

## Abstract

**Background:**

Treatment with Peginterferon Alpha-2b plus Ribavirin is the current standard therapy for chronic hepatitis C (CHC). However, many host related and viral parameters are associated with different outcomes of combination therapy.

**Objectives:**

The aim of this study was to develop an artificial neural network (ANN) model to predetermine individual responses to therapy based on patient’s demographics and laboratory data.

**Patients and Methods:**

This case-control study was conducted in Tehran, Iran, on 139 patients divided into sustained virologic response (SVR) (n = 50), relapse (n = 50) and non-response (n = 39) groups according to their response to combination therapy for 48 weeks. The ANN was trained 300 times (epochs) using clinical data. To test the ANN performance, the part of data that was selected randomly and not used in training process was entered to the ANN and the outputs were compared with real data.

**Results:**

Hemoglobin (P < 0.001), cholesterol (P = 0.001) and IL-28b genotype (P = 0.002) values had significant differences between the three groups. Significant predictive factor(s) for each group were hemoglobin for SVR (OR: 1.517; 95% CI: 1.233-1.868; P < 0.001), IL-28b genotype for relapse (OR: 0.577; 95% CI: 0.339-0.981; P = 0.041) and hemoglobin (OR: 0.824; 95% CI: 0.693-0.980; P = 0.017) and IL-28b genotype (OR: 2.584; 95% CI: 1.430-4.668;P = 0.001) for non-response. The accuracy of ANN to predict SVR, relapse and non-response were 93%, 90%, and 90%, respectively.

**Conclusions:**

Using baseline laboratory data and host characteristics, ANN has been shown as an accurate model to predict treatment outcome, which can lead to appropriate decision making and decrease the frequency of ineffective treatment in patients with chronic hepatitis C virus (HCV) infection.

## 1. Background

Hepatitis C virus (HCV) infection is widespread, with an estimated 170 to 180 million individuals infected worldwide and 3–4 million new HCV infections each year (1, 2). The current standard of care in chronic HCV hepatitis is the combination of pegylated interferon (PEG-IFN) and ribavirin (RBV) (3-5), which around 50-60% of patients are responders to this therapy (6). However, treatment response rates differ significantly among infected patients.

A wide variety of predictors are advocated for pretreatment evaluation of response to PEG-IFN plus RBV therapy (5). For instance, while up to 80% of patients with genotypes 2 and 3 infection can be cured, the response rate is only 40–50% in genotype 1 infection. Moreover, patients aged < 40 years experienced higher rates of sustained virologic response (SVR) than those aged > 40 years. Other factors such as viral load and body mass index (BMI) can also affect the response rate to the standard treatment (4, 7-9).

There is a high risk of disease progression to liver cirrhosis and subsequently to hepatocellular carcinoma in patients with an unfavorable therapeutic response or in non-responders (3). It is beneficial to predict the response of patients to PEG-IFN and RBV combination therapy before starting the treatment because therapy can be long, costly, and with many side effects (10).

Previous investigators have used artificial neural network (ANN), as artificial intelligence paradigms, to provide a reliable outcome for clinical problems (11-14). ANN is a mathematical model,which is inspired by biological nervous system. It is composed of simple elements operating in parallel. As in nature, connections between elements largely determine the network function. ANNs recognize complex patterns between inputs and outputs via the learning process. Once the hidden association between input and output has been learned, an ANN can correctly predict output from a given input. The capability of neural networks is due to their special features including nonlinear, adaptive, and parallel processing.

## 2. Objectives

The aim of this study was to develop an ANN model based on viral and host factors to predict treatment outcomes with PEG-IFN and RBV for each patient.

## 3. Patients and Methods

### 3.1. Patients

This was a case-control study conducted in Tehran, Iran, on all patients with chronic HCV infection who referred to Tehran Hepatitis Center, Baqiyatallah Research Center for Gastroenterology and Liver Diseases between July 2005 and March 2011. One hundred and thirty nine patients of 155 patients (109 males and 30 females) were included. All patients had a previous combination therapy with PEG-IFN α2b (180 µg weekly) plus RBV (1000 mg daily) for 48 weeks and undergone liver biopsy prior to or during the treatment. In general, patients were divided into three groups based on their individual response to combination therapy (15) as follows:

1. SVR (50 patients) Representing patients with negative result for detecting RNA of HCV in blood serum at the end of treatment and at least 24 weeks after cessation of therapy;
2. Relapse (50 patients), representing patients with negative viral load results at the end of treatment and recurrence of positive result of detecting the same previous HCV RNA genotype less than 24 weeks after discontinuing the therapy;
3. Non-Response (39 patients), representing patients who had never negative viral load results or less than 2 log decrease in HCV RNA in serum during the treatment.

Patients were excluded in case of coinfections such as human immunodeficiency virus (HIV), hepatitis B virus (HBV) or other liver diseases such as autoimmune chronic hepatitis and Wilson’s disease. Patients with thalassemia and those on hemodialysis were excluded, because they received PEG-IFN as monotherapy. Finally, 16 patients were excluded.

### 3.2. Predictive Variables

To predict the individual response of each patient to chronic hepatitis C (CHC) drug therapy, some viral and host-related factors were selected which their impression on different responses to treatment in HCV infected patients had been proven in former studies. Demographic information (including age, gender, weight, and height) was collected by means of a questionnaire. BMI was calculated as weight (kilograms) divided by height (meters) squared. Patient’s blood samples were taken at the beginning of treatment. Complete blood count, fasting blood sugar, lipid profile (cholesterol and triglyceride), prothrombin time, aspartate transaminase (AST) and alanine transaminase (ALT) values were measured by valid clinical laboratories. Total biopsy score of liver histopathologic feature (using modified histology activity index (ISHAK) scoring system), quantification and qualification of HCV RNA (by reverse transcriptase polymerase chain reaction (RT-PCR) and Amplicor analysis with limit of detection 50 IU/mL), genotype of HCV (by Trugene HCV SNC genotyping assay) and genotype of IL-28B SNP (by polymerase chain reaction-restriction fragment length polymorphism (PCR-RFLP)) were determined for each patient.

### 3.3. ANN

**Figure 1.**
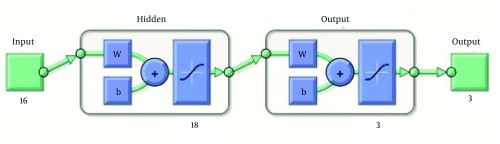
ANN’s Schematic Diagram, Consistsof Hidden and Output Layers to Receive Data From Given Inputs and Generate Outputs as Predictions

MATLAB R2010b (The Math Works, Inc.3 Apple Hill Drive, Natick, MA 01760-2098, USA) software was used to design ANN by utilizing pattern recognition tool of neural network toolbox to classify inputs into a set of target categories. The standard network used for pattern recognition is a two-layer feed-forward network, with sigmoid transfer functions in both the hidden layer and the output layer. The number of input neurons was 16, equal to the number of factors assessed for each patient in this study, respectively. Input factors are listed in Table 1. The number of neurons in hidden layer was set to 18, which the network was performed through it as well as we expected; and the number of output neurons was set to 3, which is equal to the number of elements in the target set (Figure 1). Data was divided into three sets using “divide block” function which randomly provides 3 sets of data with equal percentage of SVR, relapse and non-response patients within each set:

1. training set (70%, 97 patients);
2. validating set (15%, 21 patients) to validate that the network is generalizing and to stop training before over-fitting;
3. testing set (15%, 21 patients) as a completely independent test of network generalization.

ANN tries to estimate an output value for the given inputs by its own and compare it with their known outputs to calculate an error value; finally minimizes these error values according to back-propagation algorithm and adjusting the weights.

**Table 1.** Characteristics of Patients in General and Separately in SVR, Relapse and Non-Response Groups ^a,b^

	Total (n = 139)	SVR (n = 50), 35.97%	Relapse (n = 50), 35.97%	No. Resp. (n = 39), 28.05%	P Value
**Host factors**					
Age, y	39.87 (12.53)	40.14	39.94	39.43	0.965
BMI, kg/m^2^	23.98 (4.96)	23.75	23.84	24.43	0.795
**Gender**					
Male	109 (78.41)	42 (84)	40 (80)	27 (69.23)	0.234
Female	30 (21.58)	8 (16)	10 (20)	12 (30.76)	
**IL28b**					
CC	44 (31.65)	19 (38)	19 (38)	6 (15.38)	
CT	68 (48.92)	24 (48)	25 (50)	19 (48.71)	0.002
TT	27 (19.42)	7 (14)	6 (12)	14 (35.89)	
**WBC, cells/µL**	8479.06 (6666.48)	6684.4	9232.8	9813.58	0.053
**Hb, g/dL**	13.86 (2.71)	15.46	13.09	12.81	<0.0001
**Plt, L/μL**	318418.70 (870807.41)	208080	271860	519569.23	0.221
**FBS, mg/dL**	98.89	94.68	96.1	107.87	0.14
**TG, mg/dL**	119.27 (52.58)	124.32	112.41	121.61	0.503
**Chol, mg/dL**	144.28 (41.23)	161.48	134.22	135.15	0.001
**PT (s)**	13.10 (1.17)	12.88	13.28	13.15	0.227
**Total Biopsy Score**	9.47 (4.43)	9	9.48	10.07	0.531
**AST/ALT**	0.87 (0.36)	0.8	0.91	0.91	0.254
**Viral factors**					
Viral Load, IU/mL	1406288.4 (3362429.25)	1590823.24	1000116.36	1690438.69	0.564
**Genotype**					
1a	95 (68.34)	33 (66)	34 (68)	28 (71.79)	0.776
1b	20 (14.38)	9 (18)	6 (12)	5 (12.82)	
3a	22 (15.82)	8 (16)	8 (16)	6 (15.38)	
4a	2 (1.43)	0	2 (4)	0	

^a^ Abbreviations: ALT; alanine transaminase, AST; aspartate transaminase, BMI; body mass index, Chol; cholesterol, FBS; fasting blood sugar, Hb; hemoglobin, Plt; platelet, PT; prothrombin time, TG; triglyceride, WBC; white blood cell.

^b^ Data are presented as No. (%).

### 3.4. Statistical Analysis

SPSS11.0 (SPSS Inc., Chicago, Illinois, USA) software was used for statistical analysis. One-way ANOVA analysis and multivariate linear regression were performed to specify significant predictive variables and the odds ratios were calculated to compare the magnitude of various risk factors for the outcomes. Confusion matrices for combination of three groups of data was generated to calculate specificity, sensitivity, positive predictive value (PPV), negative predictive value (NPV), likelihood ratio positive (LR+), likelihood ratio negative (LR-) and accuracy. Significance was defined at the level of P < 0.05.

## 4. Results

Our study was performed on 139 patients with chronic HCV infection (109 men and 30 women). The mean age was 39.87±12.53. There were 50 cases (35.97%) of SVR, 50 (35.97%) of relapse and 39 (28.06%) of non-responder. The demographic characteristics of the patients are shown in Table 1. There were significant differences between the three groups regarding hemoglobin (P < 0.001), cholesterol (P = 0.001) and IL-28b genotype (P = 0.002). Table 2 shows the proportion of each output category for different genotypes of IL-28b, which indicates the role of protective C-Allele in favorable response to combination therapy. Patients carrying CC and CT alleles are more likely to have at least an initial response to treatment. Multivariate linear regression analysis was used to detect the significant predictive factors of SVR, relapse and non-response. As shown in Table 3, the predictive factor of SVR included hemoglobin (OR: 1.517; 95% CI: 1.233-1.868; P < 0.001), the predictive factor of relapse included IL-28b genotype (OR: 0.577; 95% CI: 0.339-0.981; P = 0.041), and the predictive factor of non-response included hemoglobin (OR: 0.824; 95% CI: 0.693-0.980; P = 0.017) and IL-28b genotype (OR: 2.584; 95% CI: 1.430-4.668; P = 0.001). There were no independent predictive factors to predetermine different responses to combination therapy.

The ANN was trained 300 times (epochs). The mean standard error was 1.2689e - 09. When the training was completed, the network output was similar to the real output. To test the ANN performance, the part of data that was selected randomly and not used in training process was entered the ANN and its output was compared with the real output. Table 4 shows the performance indices of ANN in predicting treatment outcome with Peg-IFN and RBV for each patient, compared to the real output. The accuracy of ANN model for predicting SVR, relapse and non-response were 93%, 90%, and 90%, respectively.

**Table 2.** Proportion of SVR, Relapse and Non-Response Groups in Different Genotypes of IL-28b ^a^

	SVR, %	Relapse, %	Non-Response, %
**CC**	43.18	43.18	13.63
**CT**	35.29	36.76	27.94
**TT**	25.92	22.22	51.85

^a^ Abbreviation: SVR: sustained virologic response.

**Table 3.** Multivariate Regression Analysis to Detect the Statistically Significant Factor for SVR, Relapsers and Non-Responders ^a^

Variables	Type of Response	P Value	Odds ratio ^b^(95% CI)
**Hb, g/dL**			
	SVR	< 0.0001	1.517 (1.233-1.868)
	Relapser	0.088	0.873 (0.744-1.023)
	Non-Responder	0.017	0.824 (0.693-0.980)
**Chol, mg/dL**			
	SVR	0.261	1.007 (0.996-1.018)
	Relapser	0.250	0.994 (0.984-1.005)
	Non-responder	0.905	1.000 (0.989-1.012)
**IL-28b**			
	SVR	0.263	0.677 (0.385-1.188)
	Relapser	0.041	0.577 (0.339-0.981)
	Non-Responder	0.001	2.584 (1.430-4.668)

^a^ Abbreviations: Chol; cholesterol, Hb; hemoglobin.

^b^ Odds ratios indicate the estimated increase in the log odds of the outcome per unit increase in the value of the exposure.

**Table 4.** The Performance of ANN in Predicting Treatment Outcome With Peg-IFN and RBV ^a^

	SVR, %	Relapse, %	Non-Response, %
**Sensitivity**	92	88	79
**Specificity**	94	92	94
**PPV**	90	86	83
**NPV**	95	93	91
**LR+**	15.33	11	13.16
**LR-**	0.08	0.13	0.22
**Accuracy**	93	90	90

^a^ Abbreviations: LR+; likelihood ratio positive, LR−;likelihood ratio negative, NPV; negative predictive value, PPV; positive predictive value.

## 5. Discussion

Prediction of response to PEG-IFN plus RBV treatment based on viral and host factors using ANN model was the aim of this study. Hemoglobin was the predictive factor of SVR, IL-28b genotype was the predictive factor of relapse, and hemoglobin and IL-28b genotype were the predictive factors of non-response outcome. The ANN model was able to predict SVR, relapse and non-response outcomes with good accuracies.

The role of many factors in different responses of patients receiving chronic hepatitis C therapy was proved in previous investigations. Among these factors, younger age, female gender, absence of obesity, favorable genotype (genotypes 2 and 3 as opposed to genotypes 1 and 4), minimal or absence of fibrosis and milder hepatitis in case of liver histopathology, low baseline HCV RNA level (< 600 000 IU/mL) were associated with remarkable better response (7-9, 16-21). In this study, there were significant differences between SVR, relapse and non-response groups with respect to hemoglobin, serum level of cholesterol and IL-28b genotype.

Using multivariate LR analysis, higher levels of hemoglobin were associated with increase in SVR rate, which is in agreement with shirakawa et al. results. They found higher pretreatment hemoglobin levels in SVR group compared to Non-SVR (10). It may be against the theory that anemia induced by CHC drug therapy (exclusively due to RBV) can improve the treatment results and occasionally lead to SVR (22). However, RBV dose reduction as a routine interventional method in such patients has been reported by dramatically lower SVR rates and prescription of erythropoiesis-stimulating agents has been shown to be a better approach to improve the general condition of patients and drug compliance (23); therefore, reduced hemoglobin level state may be only an indicator of patient’s better corporal response to medication which increases the chance of SVR.

The level of total cholesterol in SVR group was higher than other groups, whereas it was not an independent predictive factor of treatment outcome. Harrison et al. reported in his retrospective study that elevated serum cholesterol levels have been associated with higher SVR rates through unknown mechanisms. However, increase in SVR rate can be due to statin use in patients with elevated cholesterol level and it needs further trials assessing potential advantages of statins as adjuvant therapy for CHC (24).

In agreement with previous studies, IL-28b genotype is a strong predictor of treatment outcome in HCV patients. The global difference of alleles frequency can explain the ethnic variations in treatment response among different populations (4, 25, 26). In the case of rs12979860 genotype, McCarthy et al. and sharafi et al. reported that patients carrying protective C-allele, had about 6-fold increase in SVR rate compared to CT and TT genotypes. According to our results, the C/C variant of the rs12979860 polymorphism was associated with an increased likelihood of SVR, whereas patients with TT genotype were more likely to be non-responders (27-29).

In former researches, patients who had undetectable HCV RNA at the end of therapy (48 weeks) considered to have SVR or named responders, and non-responders have been classified as patients whom HCV RNA counting did not suppress to undetectable at the end of treatment (11, 12). Apart from these, in responders group, if HCV RNA becomes detectable again at week 24 after cessation of therapy, patient is considered to have relapsed. It is important to differentiate sustained virologic responders and relapsers, because relapsers may profit from longer courses of treatment or retreatment recommendations. Therefore, dividing the data into three SVR, relapse and non-response categories and using IL-28b SNPs polymorphism in the set of inputs made this study unique and validated the results.

In earlier studies, logistic regression (LR) models were mainly used as a non-invasive, technical method to predict treatment outcomes (30-32). On the other hand, in some articles the performance differences between two LR and ANN models were discussed in which ANN showed a significantly better performance (12, 33). Considering all these cases, an ANN model was designed which is a non-linear statistical data modeling tool. ANN has the benefit of being able to learn non-linear interconnectivity of inputs and correlations between inputs and outputs by using a set of observations and put them into continuous functions to generate an accurate predictive model without the need of understanding the underlying relationships (13, 14, 34).

Results and calculated performance parameters for each output category showed that designed ANN was able to develop an accurate, non-invasive and effective method, which can be applied on computer-based models for clinical purposes, receiving routine and inexpensive pretreatment clinical data of CHC infected patients and estimating the final response to treatment. The small number of entrance data (especially non-responders group) may be responsible for subsided accuracies and modeling could be extended using additional groups of data. This model should be validated in other populations before clinical implementation. By using such pretreatment predictive strategies in health and medical services, we can obviously reduce the number of patients who may undergo a course of treatment with potential side effects from which they would not drive a benefit. In conclusion, planning a predictive model based on simple and routine laboratory data, by utilizing the ANN, could clearly provide an estimation of how patients respond to PEG-IFN plus RBV therapy, which would be expected to be applied in interventional decision-making.
